# Metabolic improvements following Roux-en-Y surgery assessed by solid meal test in subjects with short duration type 2 diabetes

**DOI:** 10.1186/s40608-017-0149-1

**Published:** 2017-03-01

**Authors:** Sudha S. Shankar, Lori A. Mixson, Manu Chakravarthy, Robin Chisholm, Anthony J. Acton, RoseMarie Jones, Samer G. Mattar, Deborah L. Miller, Lea Petry, Chan R. Beals, S. Aubrey Stoch, David E. Kelley, Robert V. Considine

**Affiliations:** 1 Experimental Medicine, Merck and Company, Rahway, NJ USA; 2 Division of Endocrinology, Department of Medicine, Indiana University School of Medicine, Indianapolis, IN USA; 3 Community Bariatric Services, Indianapolis, IN USA; 4 Department of Surgery, Oregon Health & Science University, Portland, OR USA

**Keywords:** Roux-en-Y, Meal test, Gut hormones, Glucose, Insulin, C-peptide, GLP-1

## Abstract

**Background:**

Glucose homeostasis improves within days following Roux-en-Y gastric bypass (RYGB) surgery. The dynamic metabolic response to caloric intake following RYGB has been assessed using liquid mixed meal tolerance tests (MMTT). Few studies have evaluated the glycemic and hormonal response to a solid mixed meal in subjects with diabetes prior to, and within the first month following RYGB.

**Methods:**

Seventeen women with type 2 diabetes of less than 5 years duration participated. Fasting measures of glucose homeostasis, lipids and gut hormones were obtained pre- and post-surgery. MMTT utilizing a solid 4 oz chocolate pudding performed pre-, 2 and 4 weeks post-surgery. Metabolic response to 4 and 2 oz MMTT assessed in five diabetic subjects not undergoing surgery.

**Results:**

Significant reductions in fasting glucose and insulin at 3 days, and in fasting betatrophin, triglycerides and total cholesterol at 2 weeks post-surgery. Hepatic insulin clearance was greater at 3 days post-surgery. Subjects exhibited less hunger and greater feelings of fullness and satisfaction during the MMTT while consuming 52.9 ± 6.5% and 51.0 ± 6.5% of the meal at 2 and 4 weeks post-surgery respectively. At 2 weeks post-surgery, glucose and insulin response to MMTT were improved, with greater GLP-1 and PYY secretion. Improved response to solid MMTT not replicated by consumption of smaller pudding volume in diabetic non-surgical subjects.

**Conclusions:**

With a test meal of size and composition representative of the routine diet of post-RYGB subjects, improved glycemic and gut hormone responses occur which cannot be replicated by reducing the size of the MMTT in diabetic subjects not undergoing surgery.

**Trial registration:**

Clinical Trials.gov Identifier: NCT00957957 August 11, 2009.

## Background

Bariatric surgery is an effective means of promoting significant long-lasting weight loss with concomitant improvement and/or resolution of diabetes and other associated co-morbidities. Of great interest is the observation that the Roux-en-Y gastric bypass (RYGB) procedure improves both fasting and dynamic measures of glucose homeostasis well before significant weight loss, resulting in the concept that RYGB surgery “cures diabetes” through mechanism(s) that are separable from weight loss itself [1–4]. As recently reviewed [5] possible weight lost independent mechanisms include the significant caloric restriction that reduces hepatic and pancreatic fat, the exclusion of nutrients from the duodenum, and the more rapid entry of nutrients into the distal small intestine with concomitant increased release of bile acids and gut hormones such as glucagon-like peptide 1 (GLP-1). While it is likely that no one mechanism is solely responsible for the acute improvements in glucose homeostasis, studies designed to test each mechanism independently often require post-surgery patients to engage in ingestive behavior that is not congruent with the intent of the surgery, such as consuming large caloric loads that match pre-surgery intake and which do not reflect the behavioral responses that occur post-surgery.

Mixed meal tolerance tests are often used to assess dynamic measures of glucose homeostasis because they are a physiologically relevant challenge that mimics the free living condition. In the bariatric literature many studies use commercially available liquid mixed meals with varied macronutrient content [6–10]. However, a liquid meal may not induce all the postprandial effects of solid meal consumption. For example, it has been argued that liquids are not as effective as solids in inducing cephalic phase responses in insulin or pancreatic polypeptide release [11, 12]. Liquids require less oral processing, have more rapid gastric-emptying and orocecal transit times [13, 14], and evoke lower expected satiation in non-bariatric subjects [15, 16]. Cognitive manipulations of energy content and portion size can also significantly influence appetitive ratings and subsequent energy intake [17–19], with the perceived energy content of a food better predicting self-reported appetitive sensations than the true energy content [20, 21]. Finally, it has been shown that the cognitive and sensory effects of food form alter ingestive behavior with oral-liquid and perceived gastric-liquid preloads eliciting greater postprandial hunger and lower fullness sensations, more rapid gastric-emptying and orocecal transit times, attenuated insulin and glucagon-like peptide 1 release, and lower ghrelin suppression than did responses after oral-solid and perceived gastric-solid treatments [22].

In the present study we utilized a solid mixed meal to examine the effect of RYGB surgery to alter the behavioral and metabolic response to caloric challenge at 2 and 4 weeks post-surgery in subjects with short duration type 2 diabetes. We also assessed acute changes in fasting glucose, lipids, betatrophin (as a marker of insulin resistance [23]) and gut hormones over the same time period. Our goal was to examine metabolic responses to caloric intake that mimicked real life conditions following RYGB surgery.

## Methods

### Subjects

Eighteen women scheduled for RYGB surgery were recruited, with 17 completing the full study. One subject was unable to complete all the required procedures due to post-operative complications immediately following surgery. Inclusion criteria were morbidly obese (BMI > 35 kg/m^2^), 20–60 years old, with documented type 2 diabetes (fasting plasma glucose > 126 mg/dl or >200 mg/dl 2 h after 75 g OGTT), with total disease duration of 5 years or less at screening visit, and HbA1c ≤ 8.5%. Diabetes was managed either by lifestyle modification (drug naïve), oral medications, or with insulin (maximum daily total dose ≤ 50 units). Injectable GLP-1 analogs or oral DPP-4 inhibitors were discontinued at least 1 week prior to pre-surgery visit. At initial contact with the surgeons’ office subjects were prescribed a low carbohydrate, whole food liver reduction diet to teach the better food choices that would be required following surgery.

Five subjects not scheduled for bariatric surgery (1 women and 4 men; BMI 39.4 ± 2.7 kg/m^2^; age 47 ± 4 y) and meeting the inclusion criteria detailed above were recruited from the general population to assess the hormonal response to two different solid test meal (pudding) volumes. Three subjects were on metformin and two subjects were drug naïve at the time of testing.

All subjects gave informed consent and the protocol was approved by the Institutional Review Boards at Indiana University-Purdue University and Community Hospitals, Indianapolis, Indiana.

### Procedures

Subjects participated in a baseline visit that occurred within the 2 weeks prior to their RYGB surgery, and visits at 3 days, 1, 2 and 4 weeks post-surgery. Anthropometrics and fasting blood samples were obtained at all visits. Meal tolerance tests were performed pre-surgery and at 2 and 4 weeks post-surgery. The identical surgical procedure was performed by two surgeons and included a 1 oz gastric pouch, 100 cm Roux and 50 cm biliopancreatic limb.

#### Mixed meal tolerance test (MMTT)

A temporary indwelling catheter for venous access and sampling was placed and kept patent with saline. Bariatric subjects were provided a 4 oz pudding (~140 ml) consisting of 52.5 g whey protein, 25.0 g carbohydrate (11.1 g sugar) and 8.8 g fat. Subjects were instructed to consume the pudding within 15 min, and to eat until they were full (subjects did not have to finish the pudding). Pudding consumed was determined by weigh back. The time zero blood sample was drawn and then the subjects began to eat. Subjects interrupted eating for ~ 30–60 s for the blood draw at 10 min. All samples were drawn by syringe and immediately placed into BD P700 inhibitor tubes kept on ice. Samples were spun at 4 °C, aliquoted and frozen on dry ice within 10 min of the draw.

In the pilot effort to examine the metabolic effect of 4 versus 2 oz of pudding, subjects not scheduled for bariatric surgery were randomly assigned in a crossover design to consume one volume of pudding at the first visit, and to consume the other volume at a second visit at least 1 week later. Blood samples were drawn and processed as above.

#### Behavioral assessments

Subjects rated their hunger, fullness, how much they could eat, satisfaction, nausea and energy level on horizontally oriented 100 mm Visual Analog Scales. Ratings were completed at baseline, 30 and 120 min during the meal test.

#### Assays

Glucose, triglycerides, cholesterol, apolipoproteins, insulin and C-peptide were measured using standard clinical analyzer methods on an ADVIA Centaur and Roche Modular system. Total GLP-1 was measured by radioimmunoassay (Millipore [Billerica, MA]; within CV = 22%, between CV = 23% at 20pM), active GLP-1 by Meso Scale Electrochemiluminescence ([Rockville, MD]; within CV = 9.3%, between CV = 3.6% at 0.7 and 1.3 pM respectively), and pancreatic peptide YY (PYY) by ELISA (Millipore [Billerica, MA]; within CV = 2.66%, between CV =6.93% at 9.7 pM). For active GLP-1, values at the limit of detection were assigned a value of 0.65, the lowest standard in the assay. Measures made on samples from the 5 subjects in the pilot effort on pudding volume were done in the Considine lab at Indiana University. Glucose was measured using a Randox Daytona Clinical Analyzer, with insulin (within CV = 3.1%, between CV = 6.0% at 8.0 μU/ml) and c-peptide (within CV = 3.4%, between CV = 9.3% at 0.4 ng/ml) measured by radioimmunoassay (Millipore; Billerica, MA). Betatrophin was measured by ELISA (Phoenix Pharmaceuticals Inc [Burlingame, CA]; within CV = 8.9% at 1 ng/ml). HbA1c was measured by HPLC in the hospital lab as part of the subject’s pre-surgery blood work.

### Statistical analysis

Values are reported as mean ± SEM. Insulinogenic index was calculated by dividing the increment in insulin or c-peptide during the first 30 postprandial minutes by the increment in glucose over the same period. Hepatic insulin clearance was estimated by the molar ratio of fasting C-peptide to insulin [24]. Total area under the curve (AUC) was calculated for all measures except gut peptides, for which incremental AUC was used. To assess the significance of changes over time, data were analyzed by repeated measures ANOVA followed by Tukey’s multiple comparison test. Friedman test followed by Dunn’s Multiple Comparison Test was used for groups with unequal variance. Analyses were conducted with GraphPad Prism 5. Statistical significance was set at *P* < 0.05.

## Results

Seventeen women (BMI 53.3 ± 3.5 kg/m^2^; age 45 ± 10 y) with well-controlled type 2 diabetes (HbA1c 6.5 ± 0.7%) completed the study. The average duration of diabetes was 3.1 ± 1.7 years. Prior to surgery 14 subjects were taking antihyperglycemic therapy (7 on metformin, 1 on sulfonylurea, 3 on pioglitazone, 1 on GLP-1 analog, 1 on DPPIV inhibitor, and 1 on insulin), with 3 subjects not on any antihyperglycemic drugs. All subjects stopped their antihyperglycemic medications prior to surgery, and none required antihyperglycemic treatment following surgery.

Subjects lost weight over the course of the study, achieving a significant reduction beginning at 1 week post-surgery (143.8 ± 9.2, 140.6 ± 9.2, 135.8 ± 9.2, 131.0 ± 8.7 kg, for pre-surgery, 1, 2, and 4 weeks respectively; *P* < 0.05). As illustrated in Table 1, there were significant reductions in fasting glucose and insulin at 3 days post-surgery that were maintained over the course of the study. Fasting C-peptide was significantly lower only at 3 days and 1 week post-surgery. Hepatic insulin clearance significantly increased beginning at day 3. Triglycerides and total cholesterol were significantly lower at 2 weeks post-surgery. HDL cholesterol was significantly reduced beginning at 1 week post-surgery but there was no significant change in LDL cholesterol.

**Table 1:** Fasting measures of glucose homeostasis and lipids

	Pre-Surgery	Post-Surgery			
		3 days	1 week	2 week	4 week
Glucose [mg/dl]	131.6 ± 9.3	102.6.6 ± 6.0*	103.1 ± 5.0*	106.9 ± 4.8*	105.5 ± 3.7*
Insulin [uU/ml]	21.2 ± 3.1	9.8 ± 1.5*	10.9 ± 1.3*	14.0 ± 1.8*	12.7 ± 1.4*
C-peptide [ng/ml]	4.2 ± 0.3	2.7 ± 0.3*	2.9 ± 0.3*	3.5 ± 0.3	3.5 ± 0.3
Hepatic Insulin Clearance	10.7 ± 0.7	14.2 ± 0.9*	13.4 ± 0.6*	13.7 ± 1.2*	14.0 ± 0.8*
Triglycerides [mg/dl]	169.5 ± 16.3		146.8 ± 8.2	127.5 ± 7.4*	129.1 ± 8.4*
Total Cholesterol [mg/dl]	178.4 ± 9.6		164.2 ± 7.0	151.5 ± 7.4*	148.6 ± 7.4*
LDL Cholesterol [mg/dl]	105.0 ± 8.6		103.9 ± 7.0	100.9 ± 8.5	88.9 ± 6.9
HDL Cholesterol [mg/dl]	39.6 ± 2.2		30.9 ± 1.5*	32.1 ± 1.1*	33.8 ± 2.2*
Apo A1 [mg/dl]	131.6 ± 5.0		106.1 ± 3.5		103.8 ± 4.9
Apo B [mg/dl]	96.4 ± 7.1		104.0 ± 5.9		85.5 ± 5.7
Betatrophin [ng/ml]	2.6 ± 0.4			1.6 ± 0.1*	

* *P* < 0.05 compared to Pre-Surgery

Fasting betatrophin was significantly reduced at 2 weeks post-surgery. There was no correlation between fasting betatrophin and fasting glucose, insulin or triglycerides at the pre- or post-surgery time points. There was also no relationship between the reduction in betatrophin at 2 weeks post-surgery and the reductions in glucose, insulin or triglycerides.

### Subjective responses to meal challenge

**Fig. 1:**
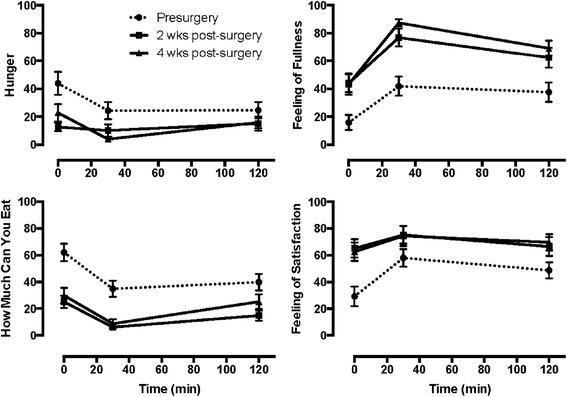
Hunger and satiety ratings during the pre- and post-surgery solid meal test. Pre-surgery AUC_total_ for each measure significantly different (*P* < 0.05) from that at 2 and 4 weeks post-surgery. See Table 2

Subjects consumed 100% of the pudding provided prior to surgery, but only 52.9 ± 6.5% and 51.0 ± 6.5% of the pudding at 2 and 4 weeks post-surgery (*P* < 0.0001). As illustrated in Fig. 1, subjects began the meal test at 2 and 4 weeks post-surgery with significantly less hunger (*P* = 0.014) and lower expectation of amount they could eat (*P* = 0.012), with greater feelings of fullness (*P* = 0.001) and satisfaction (*P* = 0.001), compared to pre-surgery. AUC_total_ for hunger and how much subjects could eat was significantly less, with that for fullness and satisfaction significantly greater at 2 and 4 weeks post-surgery (Table 2). Nausea was low with energy level moderate and not different during the three meal tests.

**Table 2:** AUC_total_ for subjective responses during meal test at 2 and 4 weeks post-surgery

	Pre-Surgery	Post-Surgery	
		2 week	4 week
Hunger	3231 ± 596	1474 ± 469*	1293 ± 283*
How Much Can You Eat	4812 ± 524	1411 ± 273*	2104 ± 1669*
Feeling of Fullness	4435 ± 706	8073 ± 658*	9002 ± 431*
Feeling of Satisfaction	6105 ± 609	8481 ± 767*	8538 ± 771*
Nausea	1218 ± 511	1771 ± 613	2078 ± 649
Energy Level	6324 ± 716	7313 ± 515	7142 ± 505

* *P* < 0.05 compared to Pre-Surgery

### Hormonal responses to meal challenge

**Fig. 2:**
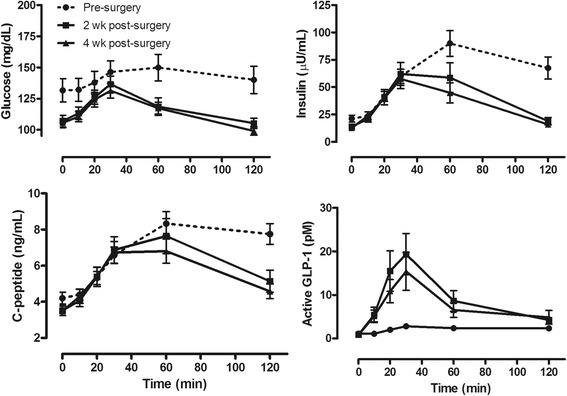
Glycemic and hormonal response to solid meal test is improved following RYGB surgery. Pre-surgery AUC_total_ for glucose, insulin and active GLP-1 significantly different (*P* < 0.05) from that at 2 and 4 weeks post-surgery. See Table 3

Fasting glucose and insulin were significantly lower at the start of the 2 and 4 week post-surgery meal test (Table 1, Fig. 2). In contrast to pre-surgery, glucose and insulin exhibited a rapid rise that peaked at 30 min, and returned to baseline by the end of the MMTT (Fig. 2). Fasting C-peptide did not differ across the three MMTT. However, during the two post-surgery MMTT, C-peptide exhibited a more parabolic response with peak at 30–60 min, and values approaching baseline by 120 min.

Insulinogenic index calculated using the change in insulin over the first 30 min was significantly lower at 2 weeks post-surgery (0.65 ± 0.22 vs 0.22 ± 0.03 nmol/mmol; *P* = 0.0095) but not at 4 weeks (0.27 ± 0.04 nmol/mmol). Insulinogenic index calculated using C-peptide was also significantly lower at 2 weeks post-surgery (1.94 ± 0.68 vs 0.73 ± 0.09 nmol/mmol; *P* = 0.0209) but not at 4 weeks (0.92 ± 0.13 nmol/mmol). AUC_total_ for glucose and insulin were significantly reduced at both 2 and 4 weeks post-surgery but C-peptide AUC_total_ was not significantly different across the study (Table 3). The ratio of AUC C-peptide/AUC glucose was not different across the study (0.32 ± 0.03, 0.32 ± 0.03, 0.30 ± 0.02 nmol/mmol). The ratio of AUC insulin/AUC c-peptide was significantly reduced at 2 and 4 weeks post-surgery compared to the pre-surgery value (8.9 ± 0.5, 6.3 ± 0.4, 5.80.4, respectively; *P <* 0.05)*.*

**Table 3:** AUC for glucose and hormone excursions during meal test at 2 and 4 weeks post-surgery

	Pre-Surgery	Post-Surgery	
		2 weeks	4 weeks
Glucose [mg/dl x min]	17,229 ± 1199	14,158 ± 666*	13,776 ± 509*
Insulin [uU/ml x min]	8027 ± 943	5142 ± 914*	4354 ± 701*
C-peptide [ng/ml x min]	857 ± 60	748 ± 81	688 ± 60
Active GLP-1 [pmol/L x min]	137 ± 29	943 ± 221*	778 ± 196*
Total GLP-1 [pmol/L x min]	1034 ± 317	3717 ± 668*	3428 ± 668*
PYY [pmol/L x min]	196 ± 160	2874 ± 643*	2058 ± 544*

* *P* < 0.05 compared to Pre-Surgery

There was little release of active GLP-1 during the pre-surgery MMTT (Fig. 2). In contrast active GLP-1 increased significantly during the two post-surgery challenges, peaking at 30 min and returning to baseline by 120 min. The incremental AUC for active GLP-1 was significantly greater at 2 and 4 weeks post-surgery as was AUC_I_ for total GLP-1. Fasting PYY was significantly reduced at 1 week post-surgery (35.3 ± 3.2 vs 23.9 ± 2.0 pmol/L; *P* = 0.001). PYY exhibited a greater AUC during the MMTT only at 2 weeks post-surgery (Table 3).

### Effect of pudding volume on metabolic response to meal test

**Fig. 3:**
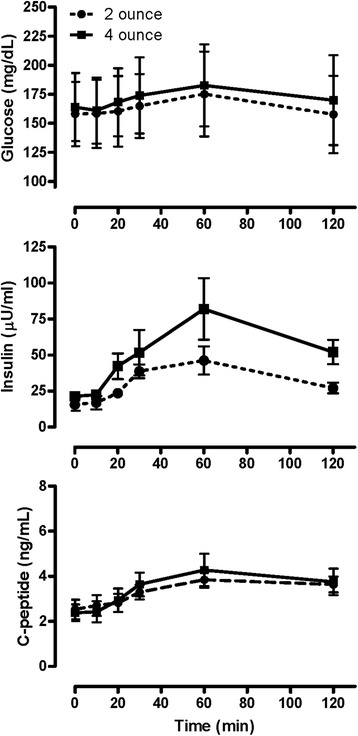
Reductions in meal size do not normalize the glycemic and hormonal response to caloric challenge in subjects with type 2 diabetes. Glucose AUC_total_ following consumption of 2 oz of pudding lower than after 4 oz (*P =* 0.016). Insulin and C-peptide AUC_total_ not significantly different by pudding size

To evaluate the effect of ingesting different pudding volumes on metabolic responses, five subjects with diabetes consumed 2 or 4 oz of pudding on separate days. As shown in Fig. 3, glucose was elevated at the start of both meal tests, and exhibited a flat response curve similar to that of the bariatric subjects pre-surgery (Fig. 2). Also as observed for the pre-surgery subjects, insulin levels peaked at 60 min and did not return to baseline at 120 min with either pudding volume. C-peptide response was the same with both pudding volumes and did not exhibit a trend for return to baseline at 120 min. Insulinogenic index calculated using the change in insulin (0.50 ± 0.26 vs 0.48 ± 0.14 nmol/mmol) or C-peptide (0.82 ± 0.4 vs 2.3 ± 1.2 nmol/mmol) was not significantly different by pudding volume. Glucose AUC_total_ was slightly less with consumption of 2 oz of pudding compared to that following 4 oz (19890 ± 3918 vs 20971 ± 4144 mg/dl x min respectively; *P = 0.016*), but remained comparable to that observed for the pre-surgery meal test. C-peptide AUC_total_ (415 ± 36 vs 443 ± 71 ng/ml x min) and insulin AUC_total_ (4153 ± 515 vs 7046 ± 154 uU/ml x min) were not statistically different by pudding volume, although the lower insulin excursion with the 2 oz meal suggests greater insulin clearance. There was no difference in AUC_total_ for hunger (5313 ± 985 vs 3981 ± 1349), how much subjects could eat (7029 ± 1639 vs 6336 ± 1680), fullness (5754 ± 1553 vs 6717 ± 1149) or satisfaction (6972 ± 1709 vs 9021 ± 739) with consumption of 2 or 4 oz of pudding. These data indicate that in the absence of surgery, consuming a meal of smaller volume does not improve the glycemic, insulin secretory or behavioral response to a test meal in obese subjects with diabetes.

## Discussion

Subjects undergoing RYGB lost a modest amount of weight over the four weeks following surgery, and none needed to resume antihyperglycemic therapy following surgery. There were significant reductions in fasting glucose and insulin, with a significant increase in hepatic insulin clearance, as early as 3 days after surgery. Fasting betatrophin, triglycerides, total cholesterol, and HDL were significantly decreased post-surgery. Subjects exhibited less hunger, lower expectation of the amount of food they could eat, and greater feelings of fullness and satisfaction during the meal test following surgery, at which they consumed ~50% less calories. Insulinogenic index and AUC for glucose and insulin were significantly lower at 2 weeks post-surgery. GLP-1 and PYY response to the meal was significantly increased post-surgery. The improvement in metabolic responses to the meal challenge following surgery could not be replicated by simply reducing meal volume in similar subjects who did not undergo surgery.

Acute reductions within the first post-operative week in fasting measures of glucose homeostasis support the suggestion that bariatric surgery ameliorates diabetes [1–4] and this effect appears to be primarily driven by changes in hepatic metabolism. Increased hepatic insulin sensitivity, decreased hepatic glucose production and increased hepatic insulin clearance have all been observed within the first post-operative month [25–28], beginning as early as 1 week following surgery [9, 29]. Several studies suggest that the improvement in hepatic metabolism is primarily due to the caloric restriction rather than the surgery per se [27, 30, 31]. However it is important to note that the degree of caloric restriction following RYGB surgery is difficult to achieve and maintain in the non-surgical population. Our data showing that subjects exhibited less hunger, lower expectation of the amount of food they could eat, and greater feelings of fullness and satisfaction in the fasting state support the efficacy of RYGB surgery to reduce caloric intake. It should be noted that the consumption of a low glycemic liver reduction diet prior to surgery was not sufficient to normalize hepatic metabolism to that attained following RYGB surgery.

Fasting triglycerides and total cholesterol significantly improved beginning at 2 weeks post-surgery, with a significant reduction in HDL at 1 week following surgery. A recent meta-analysis of data from 7815 subjects that underwent RYGB surgery found that, although heterogeneity among the studies included was high, triglycerides were significantly reduced by 3 months, total cholesterol and LDL by 1 month, and that HDL was unchanged until an increase at 1 year post-surgery [32]. The acute improvement in lipids observed here can likely be attributed to both reduced food intake, and reduced lipid absorption [33–35].

Betatrophin, or angiopoietin-like protein 8, is expressed in human liver and adipose tissue, is present in the circulation, and is involved in triglyceride metabolism [36]. Preclinical studies had suggested that betatrophin promoted β-cell proliferation [37] but this work has recently been retracted [38]. Betatrophin has also been suggested to be a marker of insulin resistance, although the nature of the relationship (positive or negative) is controversial, likely due to small differences in circulating protein across populations of different adiposity or diabetic status, and to differences in the commercially available human betatrophin assays [23]. In the current study we observed a small but significant reduction in fasting betatrophin at 2 weeks post-surgery, suggesting that betatrophin is increased in the diabetic state and reduced with normalization of insulin levels, although there was no correlation between betatrophin and fasting glucose, insulin or triglycerides. It is important to note that the assay used here employs an antibody targeted to the carboxy terminus of betatrophin, which has been shown to detect both full length and carboxy-terminal cleavage fragments of the protein [23]. Thus the reduction in betatrophin observed here is unlikely to be due to increased degradation of protein. A recent study has shown that insulin infusion increases adipocyte betatrophin expression in humans [39]. Thus it is possible that the reduction in circulating insulin following bariatric surgery decreases the signal for betatrophin production in adipose tissue.

In response to solid meal test following surgery, subjects experienced less hunger, lower expectation of the amount of food they could eat, and greater feelings of fullness and satisfaction, coincident with consumption of only 50% of the meal provided. Given that the volumes of liquid meal tests used in the bariatric literature range from 100 to more than 400 mls [5–9 and others] it is note-worthy that subjects in the current study only comfortably consumed about 70 mls following surgery. Factors that likely contributed to this outcome include the high protein content of the pudding, the slower more deliberate act of eating the pudding with a spoon, and individual perception that the pudding would be “filling”. These important characteristics contribute to the success of bariatric surgery to limit caloric intake, and support dietary recommendations for this population to limit beverage intake to low or no calorie liquids [40].

Insulinogenic index, a dynamic measure of glucose homeostasis was significantly lower at 2 weeks post-surgery. Insulinogenic index has been shown to reflect meal size in normal weight subjects, doubling with a two fold increase in Kcal ingested [41]. We did not observe a difference in insulinogenic index in the non-surgical diabetic subjects consuming the two different pudding volumes, suggesting that the insulin secretory response was not properly responsive to glucose load in those subjects. Our observations suggest that following surgery the dynamic response to glucose ingestion was improved.

Greater post- than pre-surgery insulin and C-peptide excursions are generally observed when consuming large volume liquid mixed meals, resulting in an increase in post-surgery insulinogenic index (for example see [8, 10]). This observation has been interpreted to reflect an improved response to glucose ingestion, although such a response is unlikely to occur given the reduction in food intake per meal following surgery. Despite a 50% reduction in caloric stimulus post-surgery, we observed similar insulin/C-peptide release over the first 30 min pre- and post-surgery, suggesting that there was an increase in insulin and C-peptide release per caloric unit consumed, likely promoted by the significant increase in secretion of GLP-1.

Insulin AUC, but not that for C-peptide, was lower during the meal test post-surgery, indicating greater insulin clearance by the liver. Reductions in fasting and dynamic hyperinsulinema contribute to improved insulin sensitivity in muscle and adipose tissue, which has been documented to occur in parallel with weight loss [42]. Lower fasting glucose and smaller glucose response to caloric challenge contribute to improved insulin sensitivity via reduced glucotoxicity in insulin target tissues. Importantly, the improvements in glucose homeostasis observed here were not achieved by simply reducing the quantity and volume of test meal ingested, as shown in the diabetic subjects that did not undergo surgery.

GLP-1 and PYY release during the meal test were greatly increased following surgery, as has been observed in a number of studies (reviewed in [43]). The enhanced secretion of GLP-1 and PYY resulting from the rapid transit of calories to the lower small intestine likely contributed to the reduction in food intake via their satiating effects on gut function and the central nervous system. The incretin effect of GLP-1 would be expected to result in greater insulin secretion during the meal test. Our observation that C-peptide release in response to the post-surgery meal test was unchanged, despite ingestion of half of the caloric load, suggests that GLP-1 did have an effect to promote greater insulin release, which was subsequently and efficiently cleared from the circulation by the liver.

A recent study in a small number of post-RYGB surgery subjects noted that a liquid meal induced greater increases in insulin and gut peptides than did a solid meal of similar caloric content [44]. This likely reflected the much faster absorption of calories provided by the liquid meal. We did not compare responses between solid and liquid meals as our intent, in contrast to that of Lee et al. [44], was not to determine a superior test method.

## Conclusions

In summary we observed significant improvements in both fasting and dynamic metabolic measures following RYGB surgery in subjects with short duration diabetes. The use of a solid mixed meal test in this study permitted demonstration of improved glycemic responses to a meal challenge under the naturalistic conditions of reduced caloric intake experienced by bariatric surgery subjects. The metabolic improvements achieved with RYGB cannot be achieved by simply reducing food intake as demonstrated in non-surgical subjects.

## Abbreviations

AUC: Area under the curve
GLP-1: Glucagon-like peptide 1
MMTT: Mixed meal tolerance test
PYY: Pancreatic polypeptide YY
RYGB: Roux-en-Y gastric bypass
